# Identifying New Clusterons: Application of TBEV Analyzer 3.0

**DOI:** 10.3390/microorganisms11020324

**Published:** 2023-01-27

**Authors:** Majid Forghani, Sergey Kovalev, Michael Khachay, Edward Ramsay, Mikhail Bolkov, Pavel Vasev

**Affiliations:** 1 N. N. Krasovsky Institute of Mathematics and Mechanics, Yekaterinburg 620108, Russia; 2 Institute of Natural Sciences and Mathematics, Ural Federal University, Yekaterinburg 620075, Russia; 3 Saint Petersburg Pasteur Institute, Saint Petersburg 197101, Russia; 4 Institute of Immunology and Physiology, Yekaterinburg 620049, Russia

**Keywords:** TBEV analyzer, monitoring, clusteron approach, epidemiology, evolution

## Abstract

Early knowledge about novel emerging viruses and rapid determination of their characteristics are crucial for public health. In this context, development of theoretical approaches to model viral evolution are important. The clusteron approach is a recent bioinformatics tool which analyzes genetic patterns of a specific E protein fragment and provides a hierarchical network structure of the viral population at three levels: subtype, lineage, and clusteron. A clusteron is a group of strains with identical amino acid (E protein fragment) signatures; members are phylogenetically closely related and feature a particular territorial distribution. This paper announces TBEV Analyzer 3.0, an analytical platform for rapidly characterizing tick-borne encephalitis virus (TBEV) strains based on the clusteron approach, workflow optimizations, and simplified parameter settings. Compared with earlier versions of TBEV Analyzer, we provide theoretical and practical enhancements to the platform. Regarding the theoretical aspect, the model of the clusteron structure, which is the core of platform analysis, has been updated by analyzing all suitable TBEV strains available in GenBank, while the practical enhancements aim at improving the platform’s functionality. Here, in addition to expanding the strain sets of prior clusterons, we introduce eleven novel clusterons through our experimental results, predominantly of the European subtype. The obtained results suggest effective application of the proposed platform as an analytical and exploratory tool in TBEV surveillance.

## 1. Introduction

Tick-Borne Encephalitis Virus (TBEV) is a causative pathogen for tick-borne encephalitis (TBE), and carries the possibility of serious neurological outcomes, including fatal ones. TBEV is an arthropod-borne virus that belongs to the *Flaviviridae* family, genus *Flavivirus*. Beginning in 2012, TBE has become one of the most notable human diseases in the European Union [1]. Annually, more than 500,000 tick bite incidents are registered in Russia, and 1500–2000 cases of TBE are reported [2]. TBEV’s geographical area of spread forms a belt starting in East Asia and ending in central Europe. Recent animal surveillance activities indicate that the virus is spreading in northern Africa, including northwestern Tunisia [3], as well as in the east/south of England [4,5]. Several of these locations, however, have not yet registered human infections.

Conventionally, TBEV has three subtypes, namely, TBEV-FE (Far Eastern), TBEV-Sib (Siberian), and TBEV-Eu (European). These have recently been increased to five by proposing two new potential subtypes: TBEV-Bkl (Baikalian) [6] and TBEV-Him (Himalayan) [7]. TBEV-Sib currently consists of four lineages: Baltic (TBEV-Sib-Balt), Asian (TBEV-Sib-Asia), South-Siberian (TBEV-Sib-S.-Sib), and East-Siberian (TBEV-Sib-E.-Sib). The subtypes can be characterized by three aspects: spatial distribution, tick vector species, and severity of pathogenicity [8]. For example, on the global scale the European subtype is transmitted by *I. ricinus*, whereas the main vector of Siberian and Far Eastern subtypes is *I. persulcatus*. TBEV-FE covers the Russian Far East and northern China, whereas TBEV-Sib is mainly encountered in northwestern Russia, the Ural region, and Siberia. TBEV-Eu is limited to Eastern, Central, and Western Europe [9]. TBEV-Bkl has been reported along the Trans-Baikal region, northern Mongolia, and the Republic of Buryatia [6]. TBEV-Him was isolated in the Qinghai–Tibet Plateau [7].

Mature TBEV features a spherical enveloped virion approximately 50 nm in diameter with a positive-sense single-stranded RNA (+ssRNA) genome. The genome, which is approximately 11 kb, encodes three structural proteins, C, precursor-M (prM), and E, along with seven nonstructural proteins, NS1, NS2A, NS2B, NS3, NS4A, NS4B, and NS5 [10]. Of the structural proteins, E protein is the major surface glycoprotein, and has specific features that have made it the focus of various studies. It is known that the overall architecture of E protein is the same for several pathogenic flaviviruses and TBEV [11]. The E protein has a substantial impact on the viral cycle. Indeed, it is one of the determinants for host-specific replication [12]. It plays roles in receptor binding, membrane fusion, virion assembly, interaction with the host immune system, and host tropism [13]. The E protein is the decisive element for phylogenetic analysis, and is a critical factor in defining virulence.

Within the endemic region, TBEV has a patchy geographical distribution [9]. A TBEV population is locally circulating, clustered, and restricted to a particular area, usually small spots. These can vary from several square meters to several square kilometers, referred to as micro- and macrofoci, respectively [14]. The migration of animals such as birds can lead to the emergence of new natural foci. Meanwhile, the natural focus environment must satisfy tick life cycle requirements, including a specific humidity rate and temperature as well as an abundance of competent hosts [15]. An extraordinary feature of TBEV is the stability of viral sequences within the natural focus; this indicates selective pressure for specific genome sequences [12]. Emerging TBEV risk areas can be identified at an early stage via surveillance of viral activity. Monitoring and evaluating TBEV endemic foci, both new and established, are essential to controlling the risk of TBEV infection in humans and to measuring focus stability dynamics [9].

In general, there are two types of monitoring platforms related to TBEV. The first type contains platforms for monitoring disease vectors, including those that transmit vector-borne diseases [16], e.g., the entomological platform specialized on monitoring ticks called VectorMap (vectormap.si.edu) [17]. The second type of platform is specialized on TBEV genetic/phylogenetic characteristics, such as the Virus Pathogen Database and Analysis Resource (ViPR) [18]. The aforementioned resources are valid and promising tools for general phylogenetic and epidemiological study. However, TBEV Analyzer provides modern fine analytical tools, such as hierarchical phylogenetic analysis performed through the clusteron approach.

The lack of a dedicated platform for studying TBEV evolution motivated us to develop the first version of the TBEV analyzer in 2019 [19]. The primary goal of the project was to create a standard, integrated, and interactive platform for TBEV population analysis based on the Clusteron Approach (CA) [6,20], yet with the ability to monitor external genetic resources, e.g., GenBank [21], for emerging novel phylogenetic strain groups and TBEV natural foci population dynamics. The word “clusteron” is derived from the terms “cluster” and “clone”. It is the smallest unit of viral population presented in the CA system. Typically, a clusteron refers to a group of strains with an identical amino acid sequence (signature) of the E protein fragment, usually related phylogenetically, and characterized by a specific type of spatial distribution [22].

The central capability of our analyzer was to provide a hierarchical analysis by inferring the phylogenetic characteristics via the CA at both global and local scales in the form of three levels, namely, subtype, phylogenetic lineage, and clusteron. The reader can refer to earlier work for concepts and more details about the CA [6,20]. Although the CA relies on several well-known bioinformatics methods (alignment, phylogenetic tree, phylogenetic network reconstruction), it requires a specific setup and configuration of algorithms to obtain reliable results. The motivation behind developing the online platform was to address this issue and to maintain reproducibility of the approach. In this way, researchers can focus on the final results without distraction by computational details.

Next, after presenting the platform’s first version, we received requests for visualization of clusteron distributions on a map. Hence, we introduced TBEV Analyzer version 2.0 in 2020 [23]. In addition to expanding previous features, the second version gained geographical mapping, customization of the alignment table to study genetic variability, and integration with GenBank for fetching the query strain. Furthermore, the user interface underwent significant changes, and many technical issues were fixed. Generally speaking, the previous versions of the platform were devoted to implementation and customization of the CA, respectively.

In this paper, we take a step forward and announce TBEV Analyzer 3.0. The main goal of the third version focuses on updating the phylogenetic model, referred to as the clusteron structure, that underlies the platform analysis. Indeed, we implemented theoretical and practical improvement to the platform. Our contributions to the latest version are as follows:

- Theoretical improvement:
  − Performing whole GenBank analysis and introducing new clusterons, followed by updating of the model of clusteron structure obtained by the clusteron approach.
- Practical improvements leading to enhancement of platform functionality:
  − Automatic monitoring of GenBank for emerging novel strains.
  − Identification of the query’s amino acid signature and its visualization on the E protein surface.
  − Provision of high-quality visualization of clusteron spatial distributions and visualization of a query on a geographical map via its latitude and longitude.
  − Interactive visualization of the clusteron structure.
  − Equipping the platform with an Application Programming Interface (API).

The remaining parts of the paper are organized as follows: Section 2 presents more details about extra features included in the latest version of the platform; further, we discuss the results obtained from analyzing the GenBank database and report new clusterons in Section 3 and Section 4. Finally, our conclusions are provided in Section 5.

## 2. Materials and Methods

As pointed out earlier, the clusteron approach is the core of our platform. The CA currently relies on performing phylogenetic analysis by constructing two phylogenetic networks [23] and having their results merged by a specialist to create a unified network called a clusteron structure (CS). At present, this procedure is carried out manually and requires several verification steps to obtain the final network of the CS. The CA can mainly be divided into two procedures:

- Constructing the CS via phylogenetic network analysis.
- Application of the CS to identify the hierarchical three-fold phylogenetic characteristics of a query strain.

The former procedure can be considered as the construction of an evolutionary model, whereas the latter is the application of the obtained model. Our project’s primary goal is to facilitate application of the CS by automatically locating the query strain in the CS graph and inferring its characteristics. Developing a unified computational pipeline for CS construction from phylogenetic networks is being considered in our future plans.

**Figure 1:**
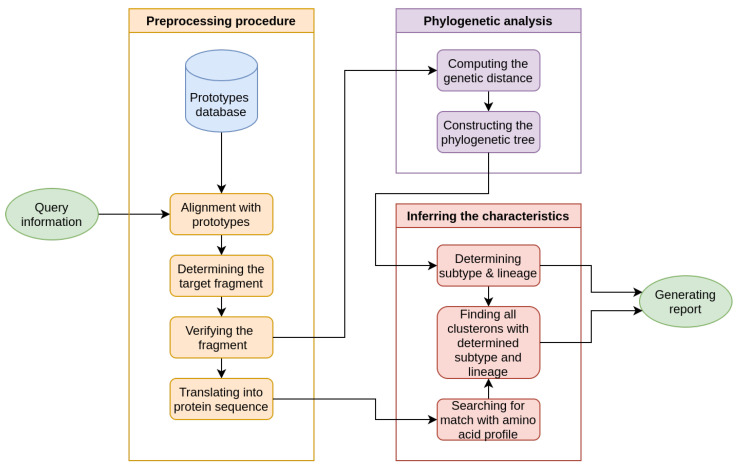
The overall schema of the computational pipeline for clusteron structure application. The pipeline generally includes alignment, identifying a target coding sequence of an E protein fragment, constructing the phylogenetic tree, and inferring/verifying the assigned clusteron to the query. Note that assigning a clusteron to a query requires verification at both the nucleotide and amino acid levels.

It is a known fact that phylogenetic analysis of TBEV strains at the nucleotide level may yield different results compared to amino acid level analysis. Hence, the CA incorporates the complementary roles of genetic information at the nucleotide and amino acid levels by combining their results. According to our computational pipeline, assigning a clusteron to a query requires verification at the nucleotide and amino acid levels. The overall schema of our pipeline was described previously [19,23], and is illustrated (with adjustment) in Figure 1.

The computational pipeline is comprised of three procedures: preprocessing, phylogenetic analysis, and inferring the characteristics. The preprocessing procedure extracts a specific E protein coding sequence fragment containing sufficient genetic information for query characterization. The fragment has a restricted length of 454 bp (from nt 309 to 762 according to the sequence of the Vasilchenko strain, GenBank: M97369). Thus, fragments with insertions, deletions, or ambiguous nucleotide characters are excluded from analysis. The second pipeline step consists of constructing a phylogenetic tree for coding sequences (called prototypes). We evaluated several tree construction algorithms and showed that this process requires knowledge about the tree on the coarse scale (i.e., clade or branch). Inferring the subtype and lineage depends on the position of the query taxon and its sister node’s clade. The specifics of the algorithm have been described elsewhere [23].

**Figure 2:**
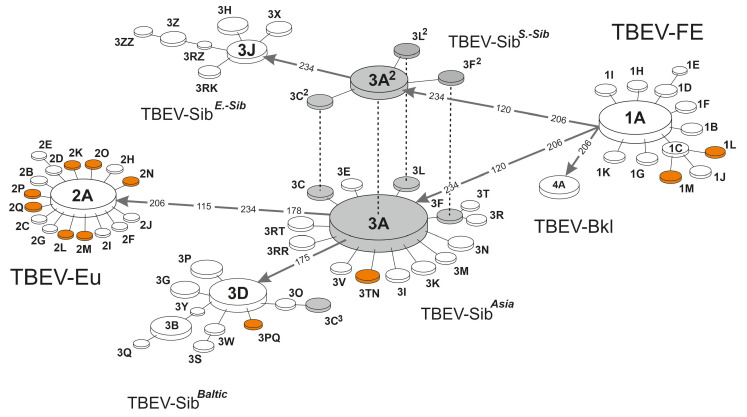
Updated version of the clusteron structure. The platform recently identified eleven new clusterons, which are highlighted in orange. Homoplastic clusterons are connected together by dotted lines.

Next, after determining the subtype and lineage, the process moves to the final step of identifying the clusteron. The decision about assigning a clusteron is taken at the amino acid level. Therefore, we search for a match in the target fragment protein sequence between the query and the clusterons using the subtype and lineage determined from the previous step. The final output is the hierarchical three-fold phylogenetic characteristics of the query strain. According to the CA, certain clusterons have identical amino acid profiles while their lineages are entirely different. This is why the amino acid sequence alone is not enough to determine the clusteron to which the query strain belongs. A dashed line visually indicates such a relationship on the CS network (Figure 2).

In case of failure to identify any of the three-fold characteristics, the strain is tagged as unique. Unique strains are viruses that do not meet the epidemiological threshold of clusteron development. In other words, in addition to the unique and specific amino acid signature, a clusteron is formed when there is enough evidence about its stability. A sole unique virus may be due to the stochastic nature of viral evolution, and its presence alone does not have epidemiological implications for the future.

This is why, in TBEV Analyzer 3.0, we implemented automatic monitoring of GenBank resources to find clues about the development of unique strains. The emergence of additional unique strains with the same profile leads to the formation of a new clusteron. However, adding a clusteron to the CS requires biological justification and verification.

The platform accepts queries containing multiple sequences, and analyzes each sequence individually to generate a report. The report consists of eight sections:

- General Report
- Clusteron Structure
- Phylogenetic Tree
- Nucleotide Alignment Table
- Protein Alignment Table
- Amino Acid Signature
- Geographical Map
- Supplementary Files

In the following sections, we briefly describe the content of each report section, including the related updates to TBEV Analyzer 3.0. The general report lists three types of information: query information entered by a user, system-generated information during task processing, and finally the three-fold phylogenetic characteristics, including the subtype, lineage, and clusteron. This is the part of the report during which a user receives the query’s brief primary features in one glance.

The “Clusteron Structure” section provides an overall picture of TBEV evolution. Such a visualization provides the opportunity to study the history of evolution at both the global and local scales. The CS assigns a unique identifier, referred to as the CS ID, to each clusteron. Typically, clusterons can be divided into two classes, the clusteron founder and the clusteron derivative [24]. The clusteron founder is a founder of a subtype/lineage, and is the greatest in number among other clusterons of a subtype/lineage. At present, there are seven founders: 1A, 4A, 3A^2^, 3A, 3J, 3D, and 2A. Clusteron derivatives are smaller, and stem from a clusteron founder. Derivatives vary based on their level (first, second, etc.). They differ from the founder by one, two, or more amino acid substitutions. The evolutionary paths between founders are decorated with ‘transition points’, i.e., amino acid sequences of the E protein fragment, that are not seen. As such, they were likely deleterious [6]. As mentioned earlier, there can be clusterons with different evolutionary paths even though their amino acid profiles are identical. Such clusterons are called homoplastic clusterons, and are connected by dotted lines (3F-3F^2^, 3A-3A^2^, 3C-3C^2^-3C^3^, 3L-3L^2^) in Figure 2. It is worth mentioning that the prototype coding sequence of a clusteron within a phylogenetic lineage is unique, even for homoplastic clusterons.

In TBEV Analyzer 3.0, we increased informativeness by adding the ability to interact with the CS. By clicking on each clusteron, a popup window displays clusteron specifics, which consist of three types of information:

- Phylogenetic characteristics
- Specific amino acid signature
- E protein fragment coding sequence (prototype)

To better visualize clusteron location, when the platform assigns a query to a known clusteron, its position in the CS blinks. With version 3.0, TBEV Analyzer gains the ability to regularly check the GenBank database and report novel emerging TBEV strains not included in the current CS. The latest analysis carried out by the platform revealed the formation of new clusterons (explained in Section 3); these were verified and added to the CS. Hence, the current CS includes the latest updated version of TBEV evolutionary dynamics.

The next report section provides the phylogenetic tree. The tree is generated from the coding sequence of the specific E protein fragment. As mentioned, the tree is necessary for declaring the subtype and lineage by comparing the query taxon’s location with clusteron taxa. More details about its algorithms and the method for determining the phylogenetic characteristics of the query have been described [23]. Note that the tree visualization is customized such that the query taxon is located as the topmost leaf in the tree. In addition, clades associated with subtypes and lineages are individually colored and the query-to-root path is highlighted in red.

Because the CA relies on both nucleotide and amino acid sequences, we provide two alignment tables equipped with several coloring schemes. The tables allow for the exploration of genetic variations among the clusterons and comparison of their signatures with the query’s signature. It should be noted that a user can examine the genetic variability from various aspects, e.g., hydrophobicity, by changing the coloring scheme. Additional information is presented as well, including information about the target fragment of the genetic sequence, position in the E protein, conserved and variable sites, and similarity score between the query and clusterons.

**Figure 3:**
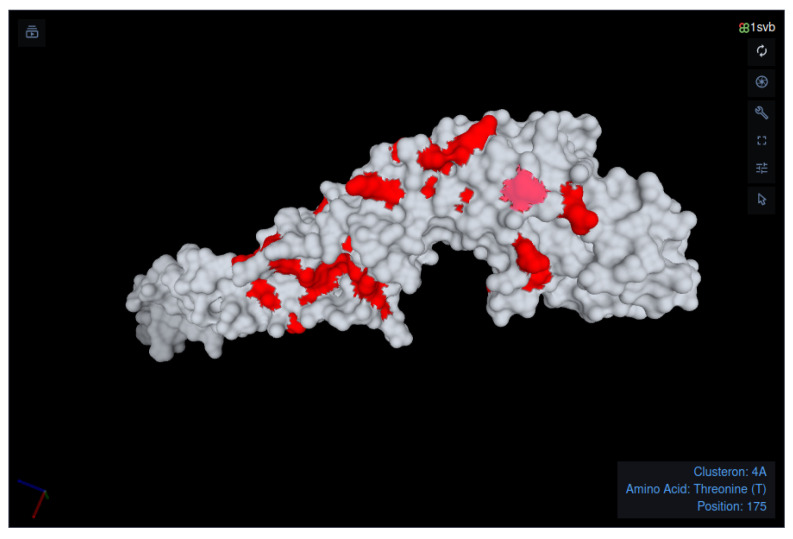
Visualization of the clusteron-specific amino acid signature on the surface of the E protein (PDB ID: 1SVB [25]). The visualization is generated by the PDBe Molstar application. The highlighted amino acids show the Baikalian subtype signature (CS ID: 4A).

Clusterons are declared based on the E protein amino acid sequence. Thus, each clusteron has its own unique specific amino acid signature, except for the homoplastic clusterons 3C-3C^2^-3C^3^, 3A-3A^2^, 3L-3L^2^, and 3F-3F^2^, which have the same signature and phylogenetically different lineages. The signatures were collected from published data [6,20] and further updated in the platform. We expanded the report by adding a new section called “Amino Acid Signature”. This section contains a table and 3D visualization. The table lists signatures of all current clusterons (Table 1), with the signature of the query’s assigned clusteron highlighted in red. Through the signatures, key sites that are responsible for phylogenetic characteristics can be determined. For example, the combination of positions 206/234 may serve as a sub-signature for subtype and lineage identification. The highlighted signature is shown on the surface of the E protein (PDB ID: 1SVB [25]) by PDBe Molstar [26,27,28] (Figure 3), a modern web-based toolkit for visualization and analysis of large-scale molecules. This type of interactive visualization permits examination of structural and functional characteristics of key protein positions.

**Figure 4:**
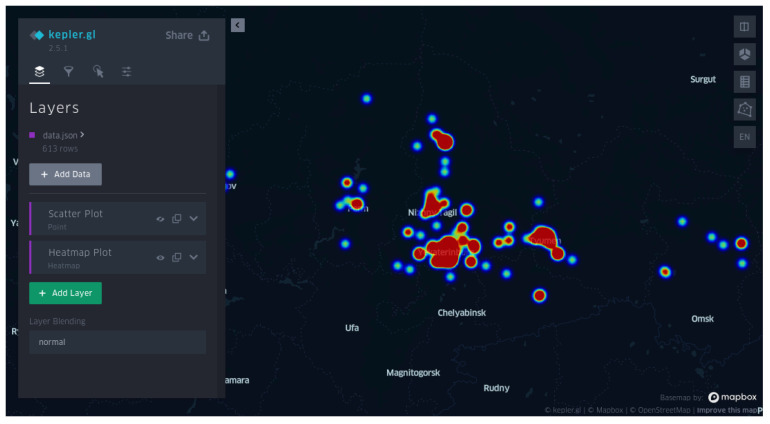
Visualization of viral distribution on the geographical map. The visualization is generated by the web application “Kepler.gl” [29]. The figure shows a heatmap of strains for clusteron 3A across the Ural region.

Compared with the second version of the platform, we visualize the TBEV spatial distribution by a high-performance WebGL-powered web application called Kepler.gl [29]. By default, the current map supports two visualizations:, a scatter plot and a heatmap. Each can be customized through the interface (Figure 4). The map is equipped with three-level filters: subtype, lineage, and clusteron. Thus, a user can personalize the map strains at each level of the CS. As a novel feature, unlike the previous version, the query strain’s location of isolation can be visualized on the map if the user provides them.

The last section of the report contains supplementary files generated by the system during query analysis. They include an aligned FASTA file of coding sequences, the phylogenetic tree file in Newick format, a phylogenetic tree image, and similarity score files for both nucleotide and protein sequences.

With the rapid growth of genetic databases, the number of online bioinformatics platforms for processing and analyzing this data has increased. The performance of platforms is enhanced via exchanging of information between them. To enable platforms to communicate, they are often equipped with an API. The current version of the TBEV Analyzer supports an API, via which the characterization of a query strain can be requested.

## 3. Experiment and Results

As mentioned earlier, TBEV Analyzer 3.0 is equipped with the remarkable advantage of regularly monitoring the GenBank database for emerging new strains. The platform performs two sequential analyses:

- Determining the three-fold phylogenitic characteristics for all new strains
- Reconsideration of the set of strains identified as unique, for finding any new clusterons

When a new strain does not belong to a known clusteron, it is tagged as a unique strain. After analyzing the GenBank database, the platform reconsiders the set of all unique strains. Suppose there are unique strains with an identical amino acid profile and their abundance meets the epidemiological threshold (e.g., minimum two strains). In such a case, they are reported and considered for further verification. When introducing a new verified clusteron into the platform database, its related unique strains are automatically labeled by its CS ID.

To demonstrate the high performance of the upgraded platform, we analyzed all TBEV-related records from GenBank. At the moment of performing this analysis, 2923 registered strains were found with the term “TBEV”. We filtered out strains with insertions, deletions, or ambiguous nucleotide characters in the region of interest. The remaining strains either had a known clusteron or were identified as unique strains. The platform analyzed 1763 strains overall, including 1419 strains with known clusterons, 46 unique strains introducing 11 novel clusterons, and 298 unique strains currently under consideration for further monitoring and analysis.

The platform found eleven new clusterons, which are marked in Table 1. Two clusterons (“1L”, “1M”) belong to the Far-Eastern subtype. Clusteron “1L” has three samples (KM019546, KJ914682, KJ739729) isolated from the Tomsk and Novosibirsk regions in Russia. Clusteron “1M” has three strains (KP869172, KF880804, KT001073) isolated from the Khabarovsk region. Seven clusterons are related to the European subtype: 2K, 2L;, 2M, 2N, 2O, 2P, and 2Q. Two remaining clusterons, 3TN and 3PQ, are associated with the Siberian subtype, with different lineages (Asian and Baltic, respectively). Characteristics of the newly analyzed clusterons are presented in Table 2.

## 4. Discussion

The emergence of COVID-19 has reminded us that many aspects of viral dynamics remain unknown and that evolution is full of surprises. Along these lines, early knowledge about antigenic variants and emerging novel viruses is crucial for public health. In addition to laboratory and clinical research, surveillance platforms play a significant role in controlling pathogens.

Although humans are an accidental host for TBEV, there is no guarantee of the virus not becoming an adapted human pathogen. The history of TBEV evolution provides strong evidence about its capability for variation in pathogenicity. Such surprising viral evolution dynamics motivated us to develop the TBEV monitoring platform.

Beginning in 2019, when we proposed a TBEV-specific platform for the first time, we have received requests to add extra features and analyses. Our efforts have envisioned the implementation of a fully automated monitoring platform with human supervision. Currently, integration of the platform with external resources, e.g., GenBank, enables it to function as a high-performance analytical tool. Newly obtained results from analyzing the GenBank database reveal the formation of novel clusterons. Essential information about their characteristics, especially their spatial distribution dynamics, was uncovered.

In addition to phylogenetic analysis, the platform presents important epidemiological information. For example, clusteron 2K, which belongs to the European subtype, was isolated in Baden-Wuerttemberg (Germany), yet it was registered in the Altai region as well. Considering the long geographical distance between Germany and Altai, a question arises about the introduction of the virus into the Altai region. A similar situation exists with clusteron 3PQ, which is related to TBEV-Sib-Baltic. Two out of four strains were found in the Omsk and Sverdlovsk regions, while the two remaining strains were collected from the Republic of Karelia, near Finland.

Other interesting facts are the spreading of TBEV in the eastern UK and reconfirmation of its origin by our platform. There are two UK-related clusterons, 2O and 2Q. Currently, clusteron 2Q covers the Netherlands and UK–Hampshire, while 2O covers Denmark, UK, and Norway. Construction of these clusterons reveals the early stage of TBEV spread in UK territory. This agrees with recent studies [4,5] that suggest introduction of TBEV from the Netherlands and Norway into the UK.

In addition to various newly added capabilities, our platform has two remarkable advantages:

- Performing CA analysis
- Monitoring the geographical distribution of clusterons

Beyond known clusteron strains, more attention should be paid to unique strains. They can be divided into two classes: evolutionary dead-end strains and evolutionary developing strains. Due to the stochastic nature of viral evolution, certain replicants acquire deleterious mutations; although they can be encountered or isolated, no further trace of them is seen in the evolutionary timeline. Because we do not have access to all isolated samples, instead of performing laboratory experiments we set an epidemiological threshold to determine the ability of a virus to develop. Unlike the deleterious mutations mentioned, selective pressure can be advantageous for a unique strain. Thus, such a unique strain may, after expansion, reach the threshold and be considered for identification as a new clusteron. In any case, all unique strains are the spotlight of the platform, and are regularly investigated algorithmically.

While the current platform is reliable and promising, there are several ways that we plan to enhance it. Thanks to the CA, we are now able to determine TBEV strain characteristics in a few seconds. The CA relies on the results of two phylogenetic networks: the first network obtained with the Median-joining algorithm [30] from phylogenetic network software developed by Fluxus (www.fluxus-engineering.com) and the second one obtained from PHYLOViZ [31], while the CS network is manually generated via merging their information. Because placing new clusterons into the CS requires reconstructing phylogenetic networks, it requires a great deal of effort. Therefore, the next stage of platform development will involve automatic CS construction.

The CS is a network presented in two dimensions. Additional characteristics, such as the age of the clusteron, can improve its informativeness. Such features will be considered in the future by producing a customized color graph in 3D space, with the additional dimension enabling the presentation of additional clusteron features.

We plan to connect the table of amino acid-specific signatures to a 3D protein surface visualization to enable exploration of differences between amino acid signatures. Thus, users will be able to interact with the table to choose positions and clusterons, allowing them to better explore genetic variability. Furthermore, by providing the degree of amino acid similarity along with reduced amino acid alphabets, researchers will be able to investigate the nature of mutations. We believe this might help aid understanding of the variability of pathogenicity within the clusterons of a lineage/subtype.

It is a known fact that there is a high degree of similarity between the E proteins of human pathogenic *Flaviviruses*. More interestingly, they have the same overall protein architecture [11]. As the CA employs a specific E protein fragment, it is possible to expand and generalize its application to other *Flaviviruses*. Furthermore, modern bioinformatics platforms include the ability to search for information within scientific papers. Therefore, we intend to develop an automated web crawler to find TBEV-related papers, look up metadata inside the web document, and fetch them.

## 5. Conclusions

In conclusion, genetic resources, especially GenBank, are accumulating more strain-specific genetic data all the time. Virologists, researchers, and other specialists need tools to bring existing voluminous data or new submissions into focus and place them in context. With newly emerging biothreats, the need to rapidly interpret data is becoming even more critical. In this paper, we propose an update to the hierarchical phylogenetic model of TBEV known as the clusteron structure, represented as a graph. Development of this version, TBEV Analyzer 3.0, includes a new comprehensive analysis of all appropriate GenBank entries for the virus. This yielded eleven new clusterons beyond those previously identified by the platform. In addition, the most recent analysis has automatically updated all clusteron strain sets, representing an important algorithmic feature of the newest version. This is an important result in that it shows that the Analyzer is capable of flagging new results while remaining consistent and reproducible with respect to previous findings. As a monitoring tool for emerging or known biological threats, this is a key requirement. In addition, other refinements were implemented for greater platform functionality. We hope that the application of the updated TBEV Analyzer can elucidate overall TBEV evolutionary dynamics or other hidden biological nuances by technological means. We foresee TBEV Analyzer and other approaches being used together to more effectively combat such viral infection and its associated health burdens.

## Figures and Tables

**Table 1:** Updated list of clusteron-specific amino acid signatures. Currently, there are 68 clusterons, which can be characterized via 34 amino acids. Note that positions 206 and 234 can represent the signature for subtype and lineage determination. The platform found eleven new clusterons (highlighted in red).

Clusteron	Prototype Strains GenBank Acc. No.	Freq. (%)	Clusteron-Specific Amino Acid Signature																																		
			115	119	120	122	126	128	130	136	137	148	151	153	158	163	165	167	169	171	175	178	188	189	192	195	201	204	206	221	228	232	234	237	239	246
TBEV-FE																																				
1A	FJ402886	100 (6.83)	T	A	S	E	K	T	H	K	I	G	V	A	S	A	F	V	S	K	T	D	V	A	V	A	E	K	S	N	K	A	N	N	E	A
1B	FJ214111	16 (1.09)	.	.	.	.	.	.	.	.	.	.	.	V	.	.	.	.	.	.	.	.	.	.	.	.	.	.	.	.	.	.	.	.	.	.
1C	DQ989336	14 (0.96)	.	.	.	.	.	.	.	.	.	.	.	.	.	.	.	.	.	.	.	.	.	.	.	.	.	.	.	.	R	.	.	.	.	.
1D	AF091013	3 (0.2)	.	.	.	.	.	.	Y	.	.	.	.	.	.	.	.	.	.	.	.	.	.	.	.	.	.	.	.	.	.	.	.	.	.	.
1E	HM008981	6 (0.41)	.	.	.	.	.	.	Y	.	.	.	.	.	.	V	.	.	.	.	.	.	.	.	.	.	.	.	.	.	.	.	.	.	.	.
1F	HM008978	3 (0.2)	.	.	.	.	.	.	.	V	.	.	.	.	.	.	.	.	.	.	.	.	.	.	.	.	.	.	.	.	.	.	.	.	.	.
1G	AB237185	2 (0.14)	.	.	.	.	.	.	.	.	.	.	.	.	.	.	.	.	.	.	A	.	.	.	.	.	.	.	.	.	.	.	.	.	.	.
1H	AF091008	8 (0.55)	.	.	.	.	.	.	.	.	.	.	.	.	.	.	.	.	.	.	.	.	.	.	.	.	.	.	.	.	.	.	.	.	.	V
1I	AY178833	5 (0.34)	.	.	.	G	.	.	.	.	.	.	.	.	.	.	.	.	.	.	.	.	.	.	.	.	.	.	.	.	.	.	.	.	.	.
1J	FJ214120	3 (0.2)	.	.	.	.	.	.	.	.	.	.	.	.	.	.	.	.	.	.	.	.	.	.	.	.	.	.	.	.	R	.	.	E	.	.
1K	X07755	14 (0.96)	.	.	.	.	.	.	.	.	.	.	.	.	.	.	.	.	.	R	.	.	.	.	.	.	.	.	.	.	.	.	.	.	.	.
1L *	KM019546	3 (0.2)	.	.	.	.	.	.	.	.	.	.	.	.	.	.	.	.	.	R	.	.	.	.	.	.	.	.	.	.	R	.	.	.	.	.
1M *	KF880804	3 (0.2)	.	.	.	.	.	.	.	.	.	.	.	.	.	.	.	.	.	.	.	.	.	.	.	.	.	.	.	.	R	.	T	.	.	.
TBEV-Eu																																				
2A	EF113081	290 (19.8)	A	.	A	.	.	.	.	.	.	.	.	.	.	.	.	.	.	.	.	E	.	.	.	.	.	.	V	.	.	.	.	.	.	.
2B	HM468162	23 (1.57)	A	.	A	.	.	.	Y	.	.	.	.	.	.	.	.	.	.	.	.	E	.	.	.	.	.	.	V	.	.	.	.	.	.	.
2C	GQ266392	8 (0.55)	A	.	A	.	.	I	.	.	.	.	.	.	.	.	.	.	.	.	.	E	.	.	.	.	.	.	V	.	.	.	.	.	.	.
2D	AJ414703	2 (0.14)	A	.	A	.	.	.	.	.	.	.	.	.	.	.	.	.	.	.	.	E	.	.	.	.	.	.	A	.	.	.	.	.	.	.
2E	HM468153	2 (0.14)	A	.	A	.	.	.	.	.	.	.	.	.	.	.	.	.	.	.	.	E	.	T	.	.	.	.	A	.	.	.	.	.	.	.
2F	AF091012	9 (0.61)	A	.	A	.	.	.	.	.	.	.	.	.	.	.	.	.	.	.	.	E	.	.	.	.	.	.	V	.	.	.	T	.	.	.
2G	EF113085	3 (0.2)	A	.	A	.	.	.	.	.	.	.	.	.	.	.	.	.	L	.	.	E	.	.	.	.	.	.	V	.	.	.	.	.	.	.
2H	TEU27495	4 (0.27)	A	.	A	.	.	.	.	.	.	.	.	.	.	.	.	I	.	.	.	E	.	.	.	.	.	.	V	.	.	.	.	.	.	.
2I	JF501438	11 (0.75)	A	.	A	G	.	.	.	.	.	.	.	.	.	.	.	.	.	.	.	E	.	.	.	.	.	.	V	.	.	.	.	.	.	.
2J	AJ319583	2 (0.14)	A	.	A	.	.	.	.	.	.	.	.	.	.	.	.	.	.	.	.	E	.	.	.	.	.	.	V	.	.	.	.	.	K	.
2K *	KC292217	9 (0.61)	A	.	A	.	.	.	.	.	.	.	.	.	.	.	.	.	.	.	.	E	.	.	.	.	.	.	V	.	.	.	.	.	.	V
2L *	DQ393776	3 (0.2)	A	.	A	.	.	.	.	.	.	.	.	.	.	.	.	.	.	.	.	E	A	.	.	.	.	.	V	.	.	.	.	.	.	.
2M *	KC154196	2 (0.14)	A	.	A	.	.	.	.	.	.	R	.	.	.	.	.	.	.	.	.	E	.	.	.	.	.	.	V	.	.	.	.	.	.	.
2N *	JQ654653	4 (0.27)	A	.	A	.	.	.	.	.	M	.	.	.	.	.	.	.	.	.	.	E	.	.	.	.	.	.	V	.	.	.	.	.	.	.
2O *	KF991107	5 (0.34)	A	.	A	.	.	.	.	.	.	.	.	.	.	.	.	.	.	.	.	E	.	.	.	.	.	.	V	.	R	.	.	.	.	.
2P *	MG210946	4 (0.27)	A	.	A	.	.	.	.	.	.	.	.	.	N	.	.	.	.	.	.	E	.	.	.	.	.	.	V	.	.	.	.	.	.	.
2Q *	LC171402	2 (0.14)	A	.	.	.	.	.	.	.	.	.	.	.	.	.	.	.	.	.	.	E	.	.	.	.	.	.	V	.	.	.	.	.	.	.
TBEV-Sib-Asia																																				
3A	AF527415	404 (27.58)	.	.	A	.	.	.	.	.	.	.	.	.	.	.	.	.	.	.	.	.	.	.	.	.	.	.	L	.	.	.	H	.	.	.
3C	GU444159	14 (0.96)	.	.	A	.	.	.	.	.	.	.	.	.	.	.	.	.	.	.	.	.	.	.	.	.	.	R	L	.	.	.	H	.	.	.
3E	GU444279	5 (0.34)	.	.	A	.	.	.	.	.	.	.	.	.	.	.	.	.	.	.	.	.	.	.	.	.	.	.	L	.	.	V	H	.	.	.
3F	GU444225	64 (4.37)	.	.	A	.	.	.	.	.	.	.	.	.	.	.	.	.	.	.	.	.	.	.	.	.	.	.	L	.	R	.	H	.	.	.
3I	GU444204	12 (0.82)	.	.	A	.	.	.	.	.	.	.	I	.	.	.	.	.	.	.	.	.	.	.	.	.	.	.	L	.	.	.	H	.	.	.
3K	EU443275	8 (0.55)	.	.	A	.	.	.	.	R	.	.	.	.	.	.	.	.	.	.	.	.	.	.	.	.	.	.	L	.	.	.	H	.	.	.
3L	GU444211	2 (0.14)	.	.	A	.	.	I	.	.	.	.	.	.	.	.	.	.	.	.	.	.	.	.	.	.	.	.	L	.	.	.	H	.	.	.
3M	GU143822	4 (0.27)	.	.	A	.	.	.	.	.	.	.	.	.	.	.	.	I	.	.	.	.	.	.	.	.	.	.	L	.	.	.	H	.	.	.
3N	AB049349	3 (0.2)	.	.	A	.	.	.	.	.	.	.	.	.	.	.	.	.	.	.	.	.	.	.	.	.	.	.	L	S	.	.	H	.	.	.
3R	KT748745	4 (0.27)	.	.	A	.	.	S	.	.	.	.	.	.	.	.	.	.	.	.	.	.	.	.	.	.	.	.	L	.	R	.	H	.	.	.
3RR	GU444227	3 (0.2)	.	.	A	.	R	.	.	.	.	.	.	.	.	.	.	.	.	.	.	.	.	.	.	.	.	.	L	.	.	.	H	.	.	.
3RT	GU444136	4 (0.27)	.	.	A	.	.	.	.	.	.	.	.	.	.	.	.	.	.	.	.	E	.	.	.	.	.	.	L	.	.	.	H	.	.	.
3T	KT749641	4 (0.27)	.	.	A	.	.	.	.	R	.	.	.	.	.	.	.	.	.	.	.	.	.	.	.	.	.	.	L	.	R	.	H	.	.	.
3TN *	MG675054	7 (0.48)	A	.	A	.	.	.	.	.	.	.	.	.	.	.	.	.	.	.	.	.	.	.	.	.	.	.	L	.	.	.	H	.	.	.
3V	FJ214137	13 (0.89)	.	.	A	.	.	.	.	.	.	.	.	.	.	.	.	.	.	.	.	.	.	.	.	.	.	.	L	.	.	.	Y	.	.	.
TBEV-Sib-S.-Siberian																																				
3A ${}^{2}$	GQ845432	50 (3.41)	.	.	A	.	.	.	.	.	.	.	.	.	.	.	.	.	.	.	.	.	.	.	.	.	.	.	L	.	.	.	H	.	.	.
3C ${}^{2}$	EF566817	2 (0.14)	.	.	A	.	.	.	.	.	.	.	.	.	.	.	.	.	.	.	.	.	.	.	.	.	.	R	L	.	.	.	H	.	.	.
3F ${}^{2}$	GQ845430	3 (0.2)	.	.	A	.	.	.	.	.	.	.	.	.	.	.	.	.	.	.	.	.	.	.	.	.	.	.	L	.	R	.	H	.	.	.
3L ${}^{2}$	EU443268	7 (0.48)	.	.	A	.	.	I	.	.	.	.	.	.	.	.	.	.	.	.	.	.	.	.	.	.	.	.	L	.	.	.	H	.	.	.
3C ${}^{3}$	JX315786	2 (0.14)	.	.	A	.	.	.	.	.	.	.	.	.	.	.	.	.	.	.	.	.	.	.	.	.	.	R	L	.	.	.	H	.	.	.
TBEV-Sib-Baltic																																				
3D	DQ393773	73 (4.98)	.	.	A	.	.	.	.	.	.	.	.	.	.	.	.	.	.	.	N	.	.	.	.	.	.	.	L	.	.	.	H	.	.	.
3B	GU444253	36 (2.46)	.	.	A	.	.	.	.	.	.	.	.	.	.	.	.	.	.	.	N	.	.	.	.	.	.	.	L	.	.	G	Q	.	.	.
3G	DQ451295	10 (0.68)	.	V	A	.	.	.	.	.	.	.	.	.	.	.	.	.	.	.	N	.	.	.	.	.	.	.	L	.	.	.	H	.	.	.
3O	GU444224	4 (0.27)	.	.	A	.	.	.	.	.	.	.	.	.	.	.	.	.	.	.	N	.	.	.	.	.	.	R	L	.	.	.	H	.	.	.
3P	FJ214139	17 (1.16)	.	.	A	.	.	.	.	.	.	.	.	.	.	.	.	.	.	.	N	.	.	.	.	.	.	.	L	.	.	.	Y	.	.	.
3Q	JX315727	5 (0.34)	.	.	A	.	.	.	.	.	.	.	.	.	.	.	S	.	.	.	N	.	.	.	.	.	.	.	L	.	.	G	Q	.	.	.
3PQ *	JX315894	4 (0.27)	.	.	A	.	.	I	.	.	.	.	.	.	.	.	.	.	.	.	N	.	.	.	.	.	.	.	L	.	.	.	H	.	.	.
3S	KT749572	6 (0.41)	.	.	A	.	.	.	.	R	.	.	.	.	.	.	.	.	.	.	N	.	.	.	.	.	.	.	L	.	R	.	H	.	.	.
3W	DQ486861	7 (0.48)	.	.	A	.	.	.	.	.	.	.	.	.	.	.	.	.	.	.	N	.	.	.	.	.	.	.	L	.	R	.	H	.	.	.
3Y	FJ214136	1 (0.07)	.	.	A	.	.	.	.	.	.	.	.	.	.	.	.	.	.	.	N	.	.	.	.	.	.	.	L	.	.	.	Q	.	.	.
TBEV-Sib-E.-Siberian																																				
3J	AB049348	24 (1.64)	.	.	A	.	.	.	.	.	.	.	.	.	.	.	.	.	.	.	.	.	.	.	.	.	.	.	L	.	.	.	Q	.	.	.
3H	AF091006	42 (2.87)	.	V	A	.	.	.	.	.	.	.	.	.	.	.	.	.	.	.	.	.	.	.	.	.	.	.	L	.	.	.	Q	.	.	.
3RK	KT749636	3 (0.2)	.	.	A	.	.	.	.	.	.	.	.	.	.	.	.	.	.	.	.	.	A	.	.	.	.	.	L	.	.	.	Q	.	.	.
3X	LC017692	7 (0.48)	.	.	A	G	.	.	.	.	.	.	.	.	.	.	.	.	.	.	.	.	.	.	.	.	.	.	L	.	.	.	Q	.	.	.
3RZ	KT321407	1 (0.07)	.	.	A	.	.	.	.	.	.	.	.	.	.	.	.	.	.	.	.	.	.	.	I	.	.	.	L	.	.	.	Q	.	.	.
3Z	KT321372	22 (1.5)	.	.	A	.	.	.	.	.	.	.	.	.	.	.	.	.	.	.	.	.	.	.	I	S	.	.	L	.	.	.	Q	.	.	.
3ZZ	KC417474	8 (0.55)	.	.	A	.	.	.	.	.	.	.	I	.	.	.	.	.	.	.	.	.	.	.	I	S	.	.	L	.	.	.	Q	.	.	.
TBEV-Baikalian																																				
4A	EF469662	17 (1.16)	.	.	.	.	.	.	.	.	.	.	.	.	.	.	.	.	.	.	.	.	.	.	.	.	.	.	L	.	.	.	.	.	.	.

*—new clusterons, identified by TBEV analyzer 3.0.

**Table 2:** Characteristics of new analyzed clusterons. The majority of them belong to European subtypes. The associated accession numbers of each clusteron are presented. The clusteron region is determined through information available in GenBank.

Subtype	Lineage	Clusteron	Strains	Region
Far-Eastern	–	1L	KJ914682 KM019546 KJ739729	Tomsk and Novosibirsk regions (Russia)
	–	1M	KP869172 KF880804 KT001073	Russia: Far East, Khabarovsk territory, Nikolaevsk and Lazo regions
Siberian	Asian	3TN	JX315908 JX315996 MF161158 MG675054 MT113363 MT113364 MT113367	Perm, Sverdlovsk and Irkutsk regions (Russia)
	Baltic	3PQ	JX315894 KT748739 MT889225 MT424734	Hizhnyi Tagil in Sverdlovsk region, Petrozavodsk and Valaam Island in Republic of Karelia, and Omsk region (Russia)
European	–	2K	KY069126 KY069125 KY069124 KT895102 KT895101 KT895100 KT895099 KT895098 KC292217	Altai region (Russia) and Baden-Wuerttemberg (Germany)
	–	2L	GU183383 DQ393776 GU183381	Estonia and Finland
	–	2M	KC154196 KC154197	Heselbach in South-East Germany
	–	2N	JQ654653 MK903681 MK903683 MK922617	(Slovenia) and Lower Saxony (Germany)
	–	2O	MN735988 MN128700 KF991107 MN735989 MN735990	Northern Zealand (Denmark), UK-Thetford Forest (United Kingdom), and Mandal (Norway)
	–	2P	AF091011 MG210946 MG210948 MG210947	Tatabanya (Hungary)
	–	2Q	MN661145 LC171402	UK-Hampshire (United Kingdom) and Netherlands

## Data Availability

TBEV analyzer 3.0 is available at https://tbev.viroinformatics.com, accessed on 1 December 2022.

## Abbreviations

The following abbreviations are used in this manuscript:

TBEV	Tick-Borne Encephalitis Virus
TBEV-FE	Tick-Borne Encephalitis Virus—Far Eastern (subtype)
TBEV-Sib	Tick-Borne Encephalitis Virus—Siberian (subtype)
TBEV-Eu	Tick-Borne Encephalitis Virus—European (subtype)
TBEV-Bkl	Tick-Borne Encephalitis Virus—Baikalian (subtype)
TBEV-Him	Tick-Borne Encephalitis Virus—Himalayan (subtype)
TBEV-Sib-Balt	Tick-Borne Encephalitis Virus—Siberian (subtype)—Baltic (lineage)
TBEV-Sib-Asia	Tick-Borne Encephalitis Virus—Siberian (subtype)—Asian (lineage)
TBEV-Sib-S.-Sib	Tick-Borne Encephalitis Virus—Siberian (subtype)—South-Siberian (lineage)
TBEV-Sib-E.-Sib	Tick-Borne Encephalitis Virus—Siberian (subtype)—East-Siberian (lineage)
CA	Clusteron Approach
CS	Clusteron Structure (subtype)
API	Application Programming Interface (subtype)
PDB	Protein Data Bank
